# Rapid microbiological diagnosis based on 16S rRNA gene sequencing: A comparison of bacterial composition in diabetic foot infections and contralateral intact skin

**DOI:** 10.3389/fmicb.2022.1021955

**Published:** 2022-10-06

**Authors:** Ying Huang, Zhizhou Xiao, Ying Cao, Fang Gao, Yingyu Fu, Mengchen Zou, Xiangrong Luo, Ya Jiang, Yaoming Xue

**Affiliations:** ^1^Department of Clinical Nutrition, The First Affiliated Hospital of Guangzhou Medical University, Guangzhou, China; ^2^Department of Clinical Nutrition, The Third Affiliated Hospital of Sun Yat-sen University, Guangzhou, China; ^3^Department of Endocrinology and Metabolism, Nanfang Hospital, Southern Medical University, Guangzhou, China

**Keywords:** 16S rRNA gene sequencing, personal genome machine, diabetic foot infections, anaerobes, tissue biopsy, swab

## Abstract

Diabetic foot infections (DFIs) represent a frequent complication of diabetes and a major cause of amputations. This study aimed to evaluate the utility of 16S rRNA gene sequencing for the rapid microbiological diagnosis of DFIs and to consistently characterize the microbiome of chronic diabetic foot ulcers (DFUs) and intact skin. Wound samples were collected by ulcer swabbing and tissue biopsy, and paired swabs of intact skin were collected from 10 patients with DFIs (five were moderately infected, and the other five were severely infected). Samples were analyzed by conventional culture and using Personal Genome Machine (PGM) 16S rRNA sequencing technology. The results showed that PGM technology detected significantly more bacterial genera (66.1 vs. 1.5 per wound sample, *p* < 0.001); more obligate anaerobes (52.5 vs. 0%, *p* < 0.001) and more polymicrobial infections (100.0 vs. 55.0%, *p* < 0.01) than conventional cultures. There was no statistically significant difference in bacterial richness, diversity or composition between the wound swabs and tissues (*p* > 0.05). The bacterial community on intact skin was significantly more diverse than that in DFUs (Chao1 value, *p* < 0.05; Shannon index value, *p* < 0.001). Gram-positive bacteria (67.6%) and aerobes (59.2%) were predominant in contralateral intact skin, while Gram-negative bacteria (63.3%) and obligate anaerobes (50.6%) were the most ubiquitous in DFUs. The most differentially abundant taxon in skin was *Bacillales*, while *Bacteroidia* was the bacterial taxon most representative of DFUs. Moreover, *Fusobacterium* (ρ = 0.80, *p* < 0.01) and *Proteus* (ρ = 0.78, *p* < 0.01) were significantly correlated with the duration of DFIs. In conclusion, PGM 16S rRNA sequencing technology could be a potentially useful technique for the rapid microbiological diagnosis of DFIs. Wound swabbing may be sufficient for sampling bacterial pathogens in DFIs compared with biopsy which is an invasive technique. The empirical use of broad-spectrum antibiotics covering Gram-negative obligate anaerobes should be considered for the treatment of moderate or severe DFIs.

## Introduction

An estimated 537 million adults aged 20–79 years worldwide have diabetes, including 140.9 million adults in China (Sun et al., 2022). Diabetic foot ulcers (DFUs) represent a serious complication of diabetes, with a reported annual incidence of 1.5–16.6% (roughly 8.3% in China) and an estimated lifetime incidence of 15–25% (Singh et al., 2005; Jiang et al., 2015; Zhang et al., 2017). It is estimated that every 20 s, a lower limb is amputated due to diabetes somewhere in the world, and nearly 90% of these amputations occur in association with infections (Lavery et al., 2006, 2007; Bakker et al., 2016). Consequently, causative organisms must be reliably diagnosed and promptly controlled. Nevertheless, existing clinical microbiology methods lack the capacity to rapidly and comprehensively diagnose complex pathogenic microorganisms in DFUs.

Traditionally, the composition of the wound microbiota has been defined using culture-based methods. However, as previous studies have indicated, conventional culture suffers from several limitations: it is time consuming (requiring 3–5 days on average), only approximately 2% of all known bacteria can be cultured in the laboratory and culture-based techniques may not necessarily reveal the most abundant or clinically important organisms *in vivo* (Rowan, 2004; Forward, 2006; Petti et al., 2006a; Dowd et al., 2008; Grice and Segre, 2011; Tuttle et al., 2011; Dunyach-Remy et al., 2014). The development of molecular techniques to identify and quantify microbial organisms has revolutionized our view of the microbial world. 16S rRNA gene sequencing may provide more definitive taxonomic classification than culture-based approaches for many organisms while also proving less time consuming and labor intensive (Petti et al., 2006b; Salipante et al., 2013). Ion Torrent Personal Genome Machine (PGM) sequencing is a cost effective and time saving technique. It has been demonstrated to be sufficiently rapid and accurate for bacterial species identification from cystic fibrosis sputum samples and periodontitis saliva samples (Sebastian et al., 2012; Salipante et al., 2013). However, the PGM technique has not yet been applied to diabetic foot infections (DFIs). Therefore, we explored the feasibility of using PGM technology for the rapid diagnosis of bacterial pathogens in infected diabetic foot wounds.

In addition, the reliability of different sampling techniques for the microbiological diagnosis of DFIs has been disputed (Pellizzer et al., 2001; Gjødsbøl et al., 2011; Lipsky et al., 2013). Most researchers consider tissue biopsy to be the most reliable sampling technique for the identification of pathogens in DFIs, but swabbing is more widely applied in clinical practice because it is easy to perform and noninvasive (Bowler et al., 2001; Gjødsbøl et al., 2011; Lipsky et al., 2013, 2020). Thus, we compared swab and tissue specimens for the microbiological diagnosis of DFIs to evaluate the necessity of performing biopsies.

Furthermore, previous studies have indicated that skin supports the growth of commensal bacteria, which directly and indirectly protect hosts from pathogenic bacteria (Chiller et al., 2001; Cogen et al., 2008). As an attempt to find a novel target for microecological prevention and to consistently characterize the microbiome of chronic DFUs, the differences in bacterial community composition between wounds and intact skin were analyzed. Moreover, Angela Oates et al. proposed that contralateral intact skin samples may provide insight into the microbial composition of skin prior to wounding because of high levels of conservation between contralateral skin sites within individuals (Oates et al., 2012). Based on this, correlation analysis of the microbiome between DFUs and contralateral intact skin was performed within individuals to provide insights into whether DFUs are vulnerable to opportunistic pathogens from skin prior to wounding.

## Materials and methods

### Patients

A total of 10 patients with DFIs were recruited for this study. All patients agreed to participate in this study and provided written consent. The study inclusion criteria were as follows: (1) a current or previous diagnosis of type 2 diabetes; (2) age ≥ 18 years; (3) the presence of a foot ulcer, which is defined as a break in the skin of the foot that involves at minimum the epidermis and part of the dermis (Van Netten et al., 2020); and (4) clinically infected DFUs, which were diagnosed based on the presence of at least two of the following symptoms: local swelling or induration, >0.5 cm of erythema around the wound, local tenderness or pain, local warmth, and purulent discharge. The severity of DFIs was graded according to the infection part of the PEDIS classification proposed by the IWGDF (Lipsky et al., 2020; Monteiro-Soares et al., 2020): grade 1 wounds were uninfected; grade 2 wounds were mildly infected, involving only the skin or subcutaneous tissue, and any erythema present extended <2 cm around the wound; grade 3 lesions were moderately infected, involving erythema extending ≥2 cm from the wound margin, and/or tissue deeper than the skin and subcutaneous tissues (e.g., bone, joint, tendon, and muscle); and grade 4 wounds were severely infected, including any foot infection with systemic inflammatory response syndrome. The study exclusion criteria were as follows: (1) receiving systemic or topical antimicrobials 2 weeks prior to this study; and (2) refusal or inability to tolerate debridement, e.g., those with severe coagulopathy, peripheral artery disease, or cardiopulmonary insufficiency.

### Sample collection

No antimicrobial agent (e.g., alcohol or iodine) or antiseptic was introduced into the wound before specimen collection. After debridement, two specimens were collected from the same area of each wound by swabbing the wound with the Levine technique (Rondas et al., 2013; S group, labeled S1, S2…S9, S10) and by taking a deep tissue biopsy (T group, labeled T1, T2…T9, T10). The Levine method involves rotating the swab over a 1 cm^2^ area of the wound for 5 s, and applying sufficient pressure to exude and collect fluid from the tissue onto the swab. Meanwhile, the area of intact contralateral skin was sampled by swabbing the skin with a cotton swab that was moistened with sterile saline (Oates et al., 2012; C group, labeled C1, C2…C9, C10). All samples were placed into sterile transport containers and transported to the laboratory within 30 min. One swab and one biopsy from the same wound were aerobically and anaerobically cultured using standard procedures. The remaining samples were frozen at −80°C until DNA extraction was performed.

### DNA extraction and PCR amplification of 16S rRNA genes

Genomic DNA was extracted from tissue and swab samples by using the QIAamp DNA Mini Kit (Qiagen). The V1–V2 hypervariable region of the 16S rRNA gene was amplified as described in the literature (Noah et al., 2008; Anne Han et al., 2011; Salipante et al., 2013). The PCR products were run on an agarose gel and purified using the AxyPrepDNA Gel Extraction Kit (Axygen). After being extracted from the gel and having their presence confirmed by further agarose gel electrophoresis, the products were quantified. Equal quantities of all samples were pooled for sequencing. Emulsion PCR was performed using the Ion Xpress Template kit V2.0 (Life Technologies) according to the instructions described in the user guide provided by the manufacturer (Sebastian et al., 2012).

### Sequencing data processing

Sequencing was carried out on the Ion Torrent PGM system (Life Technologies) using 318 chips (Salipante et al., 2013). After sequencing, the data were optimized and denoised. The resulting sequences met the following criteria: (1) contained the reverse primer and the barcode; (2) ≥ 150 bp in length; (3) no ambiguous bases; (4) homopolymers <8 bp; and (5) average quality score > 25 (Wang et al., 2015). Subsequently, we clustered the sequences into 97% similarity operational taxonomic units (OTUs) as a basis for further analysis. Finally, each processed 16S rRNA gene sequence was aligned with the SILVA rRNA database, to identify the most similar bacterial taxon with more than 80% confidence.

### Statistical analysis

Variables were compared using Student’s *t*-test (to compare normally distributed quantitative variables between two groups), one-way ANOVA (to compare normally distributed quantitative variables among three groups), or the *χ*^2^ test (to compare categorical variables). Spearman correlation analysis was used to analyze the correlations between the genera and clinical indicators of DFI patients. A *p* value of 5% was considered statistically significant (*p* < 0.05).

## Results

### Clinical data and conventional culture results

Ten patients (seven males and three females) with DFIs were enrolled in this study. Among the 10 DFUs, 5 (50%) were moderately infected, and the other 5 (50%) were severely infected. A total of 14 isolates from six genera were cultured from wound swabs, while 17 isolates from 10 genera were cultured from deep tissue specimens. The most frequently occurring genera in swab specimens and tissue specimens were *Streptococcus* (28.6%) and *Enterococcus* (23.5%), respectively. There were no obligate anaerobes (Table 1).

**Table 1:** Clinical data and conventional culture results.

Characteristics of patients and wounds						Conventional culture	
Patients	Age	Gender	Diabetes duration (years)	Ulcer duration (days)	PEDIS Grade	Swab (S Group)	Tissue (T Group)
1	65	Female	7	90	3	*Streptococcus anginosus*	*Stenotrophomonas maltophilia*
							*Staphylococcus aureus*
							*Enterococcus faecalis*
2	72	Male	16	30	3	*Staphylococcus haemolyticus*	*Enterococcus faecalis*
						*Streptococcus agalactiae*	
3	43	Male	3	30	4	*Corynebacterium*	*Corynebacterium*
							*Serratia marcescens*
4	39	Male	1	30	4	*Enterobacter aerogenes*	*Enterobacter aerogenes*
							*Citrobacter freundii*
5	68	Male	10	30	3	*Corynebacterium striatum*	*Staphylococcus haemolyticus*
						*Enterococcus faecalis*	*Morganella morganii*
6	53	Male	8	60	3	Negative	Negative
7	62	Male	16	120	4	*Staphylococcus aureus*	*Escherichia coli*
						*Proteus mirabilis*	*Proteus mirabilis*
8	62	Female	0.5	10	4	*Enterococcus avium*	*Enterococcus avium*
						*Streptococcus agalactiae*	*Enterococcus faecalis*
							*Escherichia coli*
9	70	Female	8	30	4	*Staphylococcus aureus*	*Staphylococcus aureus*
10	52	Male	10	90	3	*Proteus vulgaris*	*Proteus vulgaris*
						*Streptococcus agalactiae*	

### Composition of bacterial communities determined by 16S rRNA sequencing

**Figure 1:**
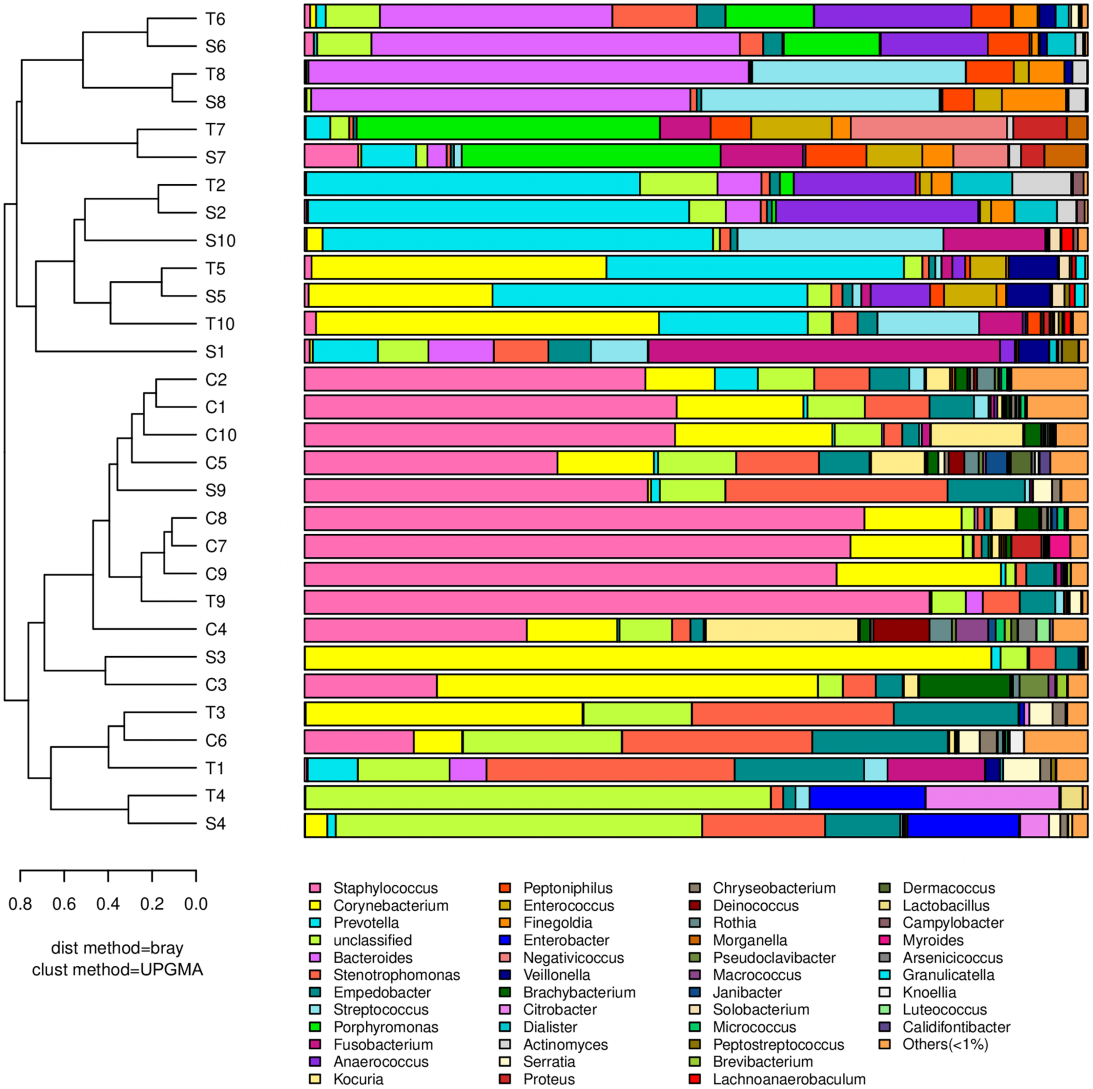
A dendrogram and histogram of the bacterial composition at the genus level. The dendrograms were constructed based on the unweighted pair group method with the arithmetic mean (UPGMA). The bacterial compositions of samples are more similar to each other with closer evolutionary relationships. The histogram visualizes the relative abundance of each sample at the genus level.

The dominant genera that had a relative abundance >1% are indicated in Figure 1. In contralateral intact skin, Gram-positive bacteria and aerobes were predominant, accounting for 67.6 and 59.2%, respectively. In diabetic foot wounds, Gram-negative bacteria (56.8% in the S Group, 63.3% in the T Group) and obligate anaerobes (54.3% in the S Group, 50.6% in the T Group) were the most ubiquitous. No significant differences in the bacterial composition of DFUs or skin were observed between females and males [permutational multivariate analysis of variance (PERMANOVA), *p* > 0.05].

*Staphylococcus* had the greatest relative abundance in contralateral intact skin (41.3%), followed by *Corynebacterium* (16.5%), *Stenotrophomonas* (6.1%), and *Kocuria* (6.0%). In diabetic foot wounds, *Prevotella* (15.5, 11.7%) was the most abundant genus in both swab and deep tissue samples, followed by *Corynebacterium* (11.9, 11.6%), *Bacteroides* (10.0, 10.9%), and *Stenotrophomonas* (6.4, 7.6%). All wound samples showed polymicrobial infections using 16S rRNA sequencing, and Gram-negative obligate anaerobes (including *Prevotella*, *Bacteroides*, *Fusobacterium*, and *Porphyromonas*) predominated in 70% of wound swabs and 50% of wound tissue samples.

### Comparison of microbiological diagnosis between conventional culture and 16S rRNA sequencing.

**Figure 2:**
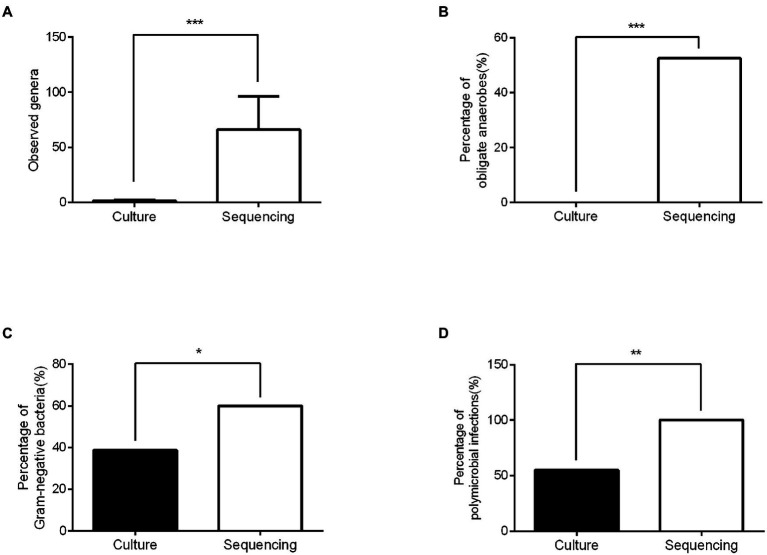
Comparison of the microbiological diagnosis between conventional culture and 16SrRNA sequencing. **(A)** The numbers of genera identified in the sequencing group were significantly higher than those in the culture group. **(B)** Significantly more obligate anaerobes were detected in the sequencing group than in the culture group. **(C)** The percentage of Gram-negative bacteria in the sequencing group was higher than that in the culture group. **(D)** More polymicrobial infections were detected in the sequencing group than in the culture group. ***p < 0.001, ** *p* < 0.01, and *p < 0.05.

16S rRNA sequencing technology detected significantly more bacterial genera (an average of 66.1 vs. 1.5 per wound sample, *p* < 0.001), more obligate anaerobes (52.5 vs. 0%, *p* < 0.001), more Gram-negative bacteria (60.0 vs. 38.7%, *p* < 0.05), and more polymicrobial infections (100.0 vs. 55.0%, *p* < 0.01) than traditional bacterial culture (Figure 2). The sequencing results contained 93.5% of genera identified by conventional culture in this study. However, 70% of the most dominant pathogens (highest abundance) diagnosed by 16S rRNA sequencing were in disagreement with conventional culture results. In addition, the bacterial culture result for DFI was negative in Patient 6, but 16S rRNA sequencing found that *Bacteroides* (Gram-negative obligate anaerobe) was dominant.

### Concordance between wound swabs and tissues analyzed by 16S rRNA sequencing

**Figure 3:**
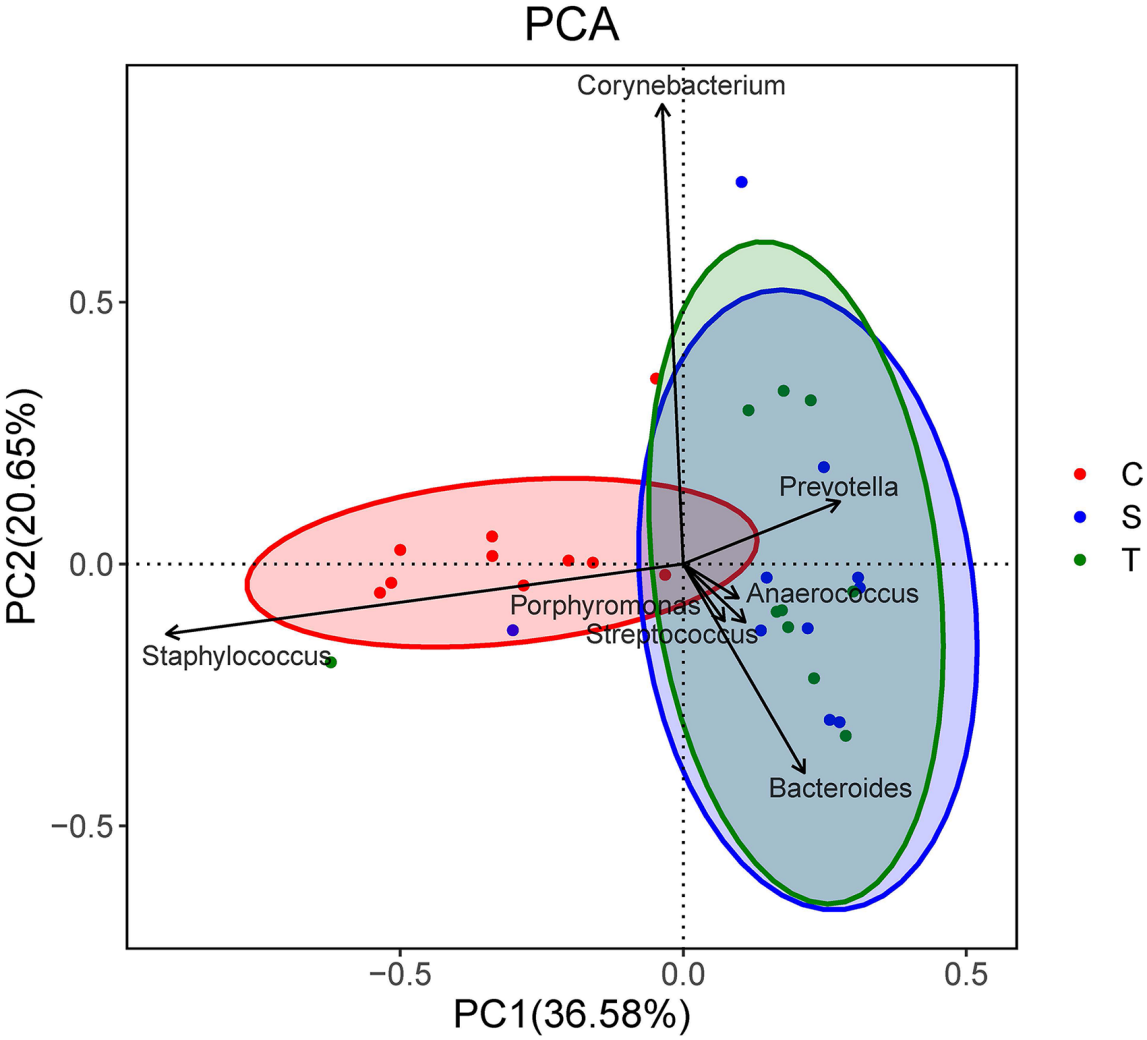
Principal component analysis (PCA) of the three groups. Samples that are more similar to each other should appear closer together along the corresponding axis reflecting the variation among all samples. Coordinates for the genera arrows are correlations with each axis, and only the seven most important (based on the magnitude of correlations) genera are shown.

**Figure 4:**
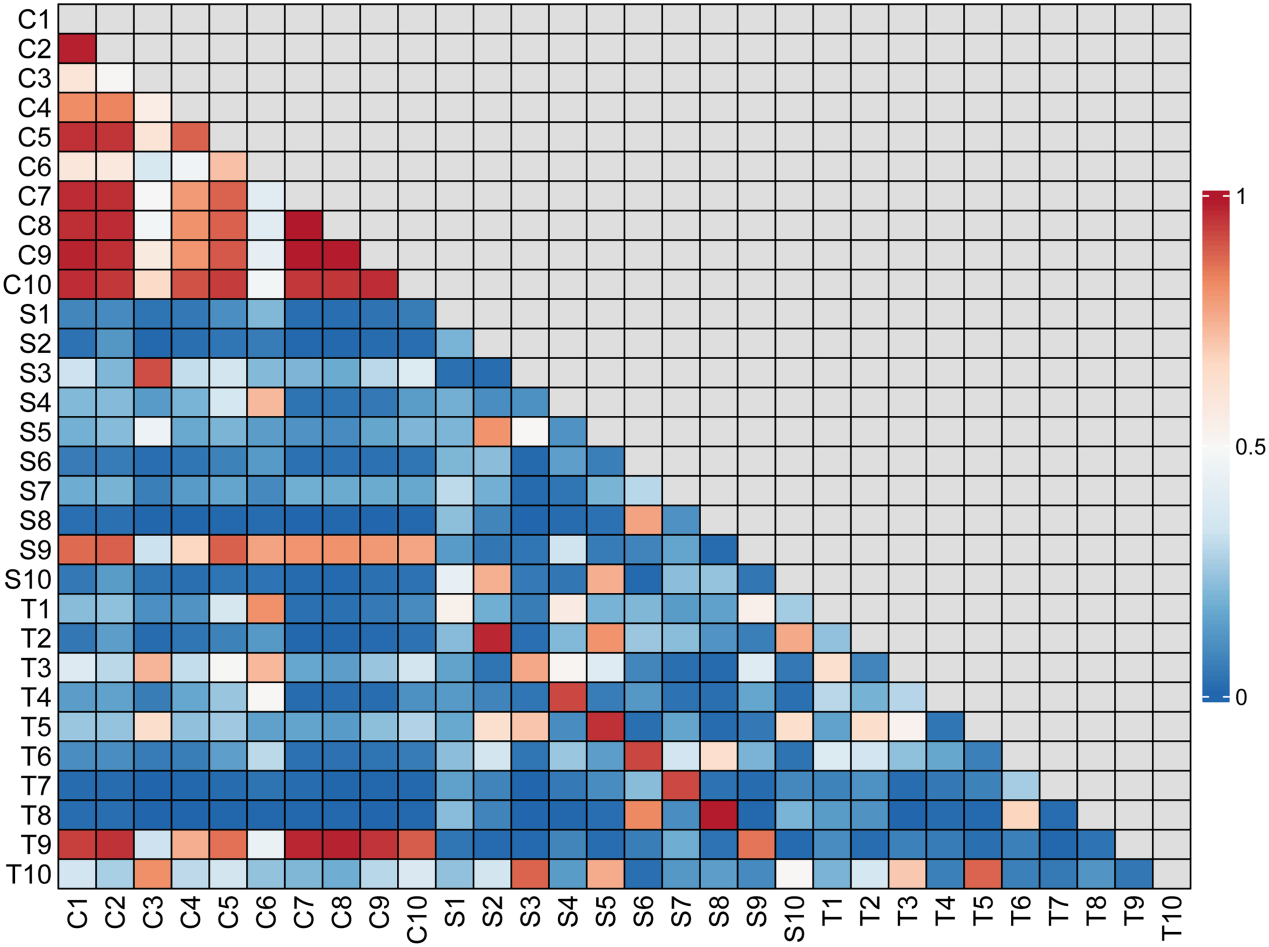
Correlation matrix for all samples. The correlation coefficient values are indicated by different shades of color according to the scale to the right of the matrix. The scale ranges from 0 to 1, where 1 represents the most similar samples.

There was no significant difference in bacterial richness or diversity between the S and T groups at the number of observed genera, Chao1 value or Shannon index value (*p* > 0.05). Subsequently, principal coordinate analysis indicated that wound swabs and tissues had similar bacterial communities (PERMANOVA, *p* > 0.05; Figure 3). Hierarchical clustering also showed that the compositions of the samples from the S Group and T Group from the same patient were similar, with noticeably closer evolutionary relationships than samples from separate patients (Figure 1). A subsequent correlation analysis revealed strong similarity between wound swabs and tissue samples within an individual, which was significantly higher than the similarity between individuals (the average correlation coefficient was 0.84 vs. 0.19, *p <* 0.001; Figure 4).

### Microbiome correlation between skin and DFUs

According to the hierarchical clustering dendrogram, there was no strong correlation of the microbiome between healthy skin and wounds within an individual in 80% of the patients; the exceptions were patients 3 and 9 (Figure 1). A subsequent correlation analysis (Figure 4) indicated significant intra-individual positive correlations between skin and DFIs in patients 3 and 9 (correlation coefficients of 0.74 and 0.95, respectively), while weak positive correlations between the paired skin and wounds were observed in the other patients (mean: 0.18). The average correlation coefficient of the C and T groups within an individual was 0.31, whereas the correlation between individuals was 0.22; the difference was not statistically significant (*p* > 0.05).

### Differences in the microbiome between DFUs and intact skin by using 16S rRNA sequencing

The bacterial richness and diversity of contralateral skin were significantly higher than those of diabetic foot wounds (including the S group and T group) in terms of the number of observed genera (*p* < 0.001), Chao1 value (*p* < 0.05), and Shannon index value (*p* < 0.001). The principal coordinate analysis indicated a significant difference in bacterial communities between diabetic foot wounds and skin (PERMANOVA, *p* < 0.01; Figure 3).

**Figure 5:**
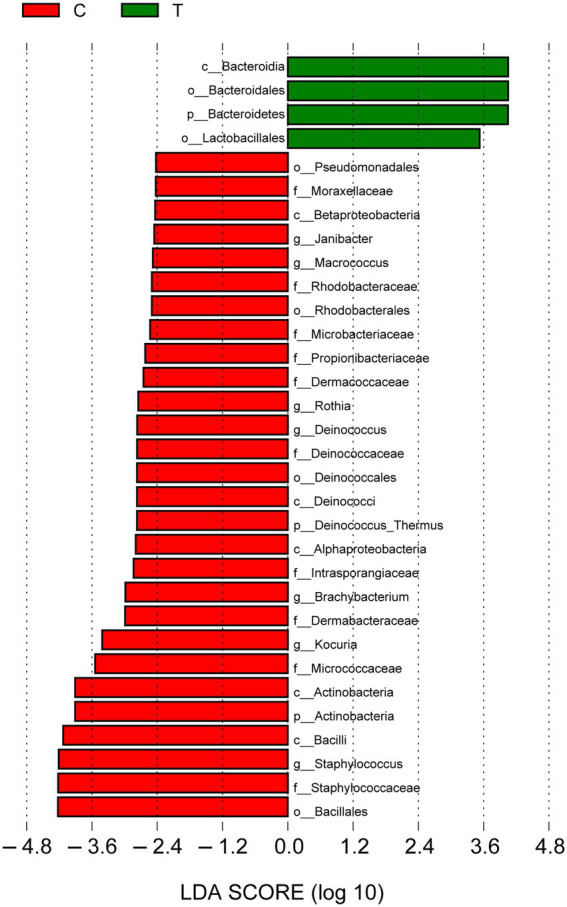
Linear discriminant analysis (LDA) effect size results for the bacterial communities of intact skin and wounds. Linear discriminant analysis indicating differentially abundant taxa between skin and wounds. Only taxa with an LDA score > 2.4 and *p* < 0.05 are shown. p_: phylum, c_: class, o_: order, f_: family, and g_: genus.

Linear discriminant analysis (LDA) effect size (LEfSe) was used to describe the effect sizes of differences in the microbiota composition between intact skin and diabetic foot wounds (Figure 5). At the phylum level, *Bacteroidetes* (LDA score = 4.05, *p* < 0.01) was enriched in DFIs, whereas *Actinobacteria* (LDA score = 3.91, *p* < 0.05) was mostly enriched in intact skin. At the genus level, no taxa in diabetic foot wounds reached the minimum LDA score, while *Staphylococcus* (LDA score = 4.21, *p* < 0.01), *Kocuria* (LDA score = 3.41, *p* < 0.001), and *Brachybacterium* (LDA score = 2.98, *p* < 0.001) were the most differentially abundant bacterial taxa in intact skin.

### Correlations between genera in DFI wounds and clinical parameters

**Figure 6:**
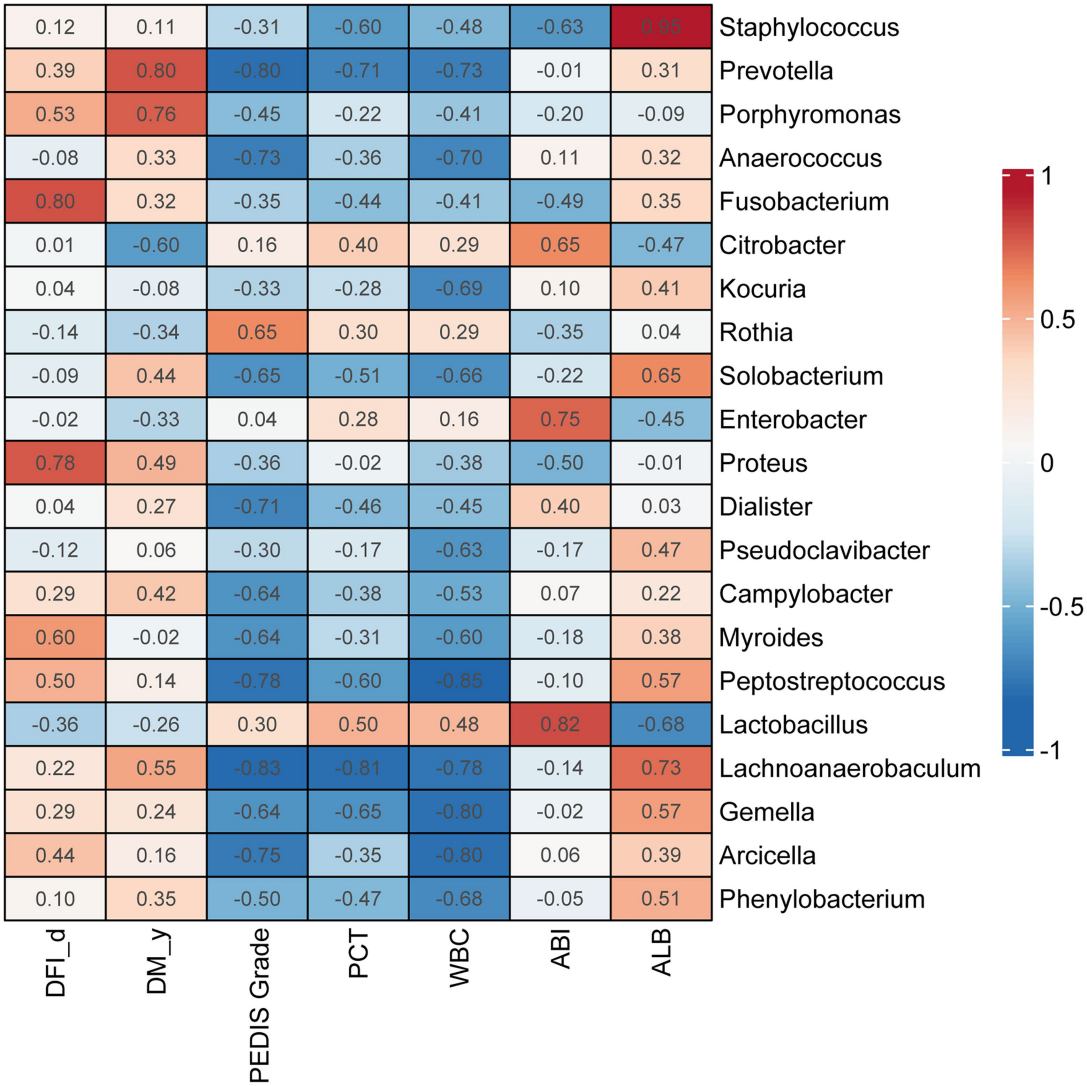
Correlations between genera in DFI wounds and clinical parameters. Correlation coefficients are marked in the heatmap. Red indicates a positive correlation with each index, and blue indicates a negative correlation with each index. DM _y: duration of diabetes (years), DFI _d: duration of diabetic foot infection (days).

*Prevotella* (ρ = 0.80, *p* < 0.01) and *Porphyromonas* (ρ = 0.76, *p* < 0.05) were significantly correlated with the duration of diabetes (DM_y). *Fusobacterium* (ρ = 0.80, *p* < 0.01) and *Proteus* (ρ = 0.78, *p* < 0.01) were significantly correlated with the duration of DFIs (DFI_d). *Rothia* was positively correlated with the PEDIS grade, and the genera with the greatest negative correlations with PEDIS grade were *Lachnoanaerobaculum* (ρ = 0.83, *p* < 0.01) and *Prevotella* (ρ = 0.80, *p* < 0.01; Figure 6).

## Discussion

A rapid and accurate technique for identifying pathogenic bacteria from infected diabetic foot wounds would be of great value in helping clinicians quickly select targeted antibiotic treatments. The results of our study highlight PGM sequencing as a promising tool for the bacteriological diagnosis of DFIs. By comparing the results of PGM sequencing and conventional culture, we identified the following advantages of 16S rRNA sequencing for identifying pathogenic bacteria in DFIs: (1) fast speed (2.3–7.3 h of running time, total process <24 h) leads to rapid diagnosis of the bacterial community, which enables the informed clinical selection of targeted antibiotics in a timely manner. (2) 16S rRNA sequencing technology detected significantly more bacterial genera and more polymicrobial infections than conventional culture. Cultures may greatly underestimate the diversity and richness of the microbiome in DFIs. (3) PGM technology is not restricted by culture conditions and can identify bacteria that have fastidious requirements for their growth environment. In this study, 16S rRNA sequencing detected that Gram-negative bacteria and obligate anaerobes were predominant in DFIs. However, the prevalence of anaerobes may be underrepresented by conventional culturing, while overestimating the abundance of Gram-positive aerobes (such as *Staphylococci*, *Streptococcus,* etc.) which grow more easily in ordinary culture medium. (4) Theoretically, 16S rRNA sequencing may better reflect the true bacterial community of the samples *in vivo* than traditional culture. Some bacteria grow more rapidly than others during the culture procedure and may come to dominate the composition of the culture; thus, the results of culture may not reflect the true bacterial composition within the sample (Moon et al., 2021). In contrast, 16S rRNA sequencing identifies the pathogenic bacteria of DFIs by processing and analyzing the genomic DNA of samples directly. Thus, the sequencing results can be largely protected from influence by the *in vitro* environment. This may be one of the reasons for the low consistency of microbiological results between conventional culture and 16S rRNA sequencing. Based on these results, PGM 16S rRNA gene sequencing may be able to increase the reliability and speed of identification of bacterial communities in wounds to provide a basis for timely and efficient antibiotic selection for patients with DFIs.

The International Working Group has proposed that clinicians should obtain cultures from a tissue specimen rather than from a swab for DFIs (Bowler et al., 2001; Lipsky et al., 2020). However, some researchers have proposed that it is sufficient to use swabbing instead of the more invasive procedure of tissue biopsy to identify pathogens, as swabs can recover high relative abundances of known and potential genera (Gjødsbøl et al., 2011; Travis et al., 2020). In our study, the sequencing results showed no significant differences in the richness, diversity, or composition of bacterial communities between the two different specimen types. The results were consistent with those of some early studies in DFUs and other chronic wounds (Pellizzer et al., 2001; Gjødsbøl et al., 2011). However, other studies showed that the quantity of pathogens or composition of bacterial communities identified by swabbing significantly differ from those obtained by tissue biopsy in DFUs (Dunyach-Remy et al., 2014; Nelson et al., 2016; Travis et al., 2020). This discrepancy might be related to the cleansing or debridement step performed before specimen collection and the Levine swabbing technique used in our study, which may reduce superficial contamination and facilitate access to exudate fluid from deeper in the wound bed by swabbing. In addition, the distribution of organisms within the wounds is patchy, which may lead to different results of bacterial communities between swab and tissue samples. In the present study, we collected swab samples and paired tissue biopsy samples from the same area of each wound, while J. Travis et al. collected tissue samples from an unswabbed area (Travis et al., 2020), and Frank et al. collected swab samples covering a greater wound surface area than the tissue biopsy (Frank et al., 2009). Biopsy to obtain tissue specimens is more invasive than swabbing and may cause damage to surrounding tissues and blood vessels. In addition, biopsy requires a skilled clinician and usually needs to be performed under local anesthesia (Gjødsbøl et al., 2011; Travis et al., 2020). Our study showed that by ulcer swabbing using the Levine technique, which is less invasive and easier to perform than biopsy, followed by 16S rRNA gene sequencing may be suitable for sampling bacterial pathogens in DFIs.

In this study, 16S rRNA gene sequencing results indicated that Gram-positive bacteria and aerobes were predominant in the intact skin contralateral to DFUs, while Gram-negative bacteria and obligate anaerobes were the most ubiquitous in infected diabetic foot wounds. Some earlier studies have also shown that anaerobic bacteria are the most prevalent pathogens in chronic wounds (Urbancic-Rovan and Gubina, 2000; Howell-Jones et al., 2005; Dowd et al., 2008). At the genus level, *Staphylococcus*, *Corynebacterium*, *Stenotrophomonas*, and *Kocuria* had the greatest relative abundance in skin, while *Prevotella*, *Corynebacterium*, *Bacteroides*, and *Stenotrophomonas* were the most abundant genera in DFUs. Notably, there are some dominant genera (such as *Corynebacterium*, *Stenotrophomonas*, etc.) shared between contralateral skin and wounds within an individual; these genera are believed to be opportunistic pathogens from skin. Further analysis indicated that patient 3 and patient 9, who were infected predominantly with *Corynebacterium* and *Staphylococcus,* respectively, showed strong intra-individual positive correlations between skin and DFI microbiome composition, reflecting the principle that opportunistic pathogens from skin sometimes may result in DFIs. However, the correlation coefficients of paired and unpaired skin/wound samples were not significantly different; thus, it cannot be concluded that an individual wound should be more similar to the corresponding skin than to any other DFI patient’s skin. This may be due to the high similarity of skin microbiomes between individuals with DFIs. Further research should be performed to compare the microbiomes of intact foot skin among people with diabetes, people with DFIs, and healthy people to better understand the correlation of the microbiome between DFIs and corresponding skin. Our study suggests that clinicians should focus on treatments that target Gram-negative obligate anaerobes and opportunistic pathogens in moderate or severe DFIs when selecting an empirical antibiotic therapy.

Our study also found that skin had significantly higher bacterial richness and diversity than diabetic foot wounds, indicating that the microecological balance of skin would be destroyed and that pathogenic bacteria would predominate instead of resident flora when the skin of diabetic patients had an ulcer. These results were consistent with the results reported by Scot E. Dowd et al. (Gontcharova et al., 2009). LEfSe analyses were performed, emphasizing statistical significance, biological consistency, and effect relevance (Segata et al., 2011; Pang et al., 2020), with the aim of identifying differential biomarkers that explain most of the effect differentiating phenotypes of DFIs and intact skin. The results demonstrated that *Bacteroidia*, *Bacteroidales*, *Bacteroidetes*, and *Lactobacillales* represented bacterial taxa in diabetic foot wounds, but no taxa at the family or genus level were consistently present because of the dispersive distribution of bacterial families and genera in DFIs. Further correlation analysis indicated that *Prevotella* and *Porphyromonas* were positively correlated with the duration of diabetes. *Fusobacterium* and *Proteus* were positively correlated with the duration of DFIs. The results were partially consistent with a previous study, reporting that the DFU duration was positively correlated with *Proteobacteria*, and the ulcer depth was associated with the abundance of anaerobic bacteria (Gardner et al., 2013). These results further emphasized the importance of performing anaerobic culture and using broad-spectrum empirical antibiotics covering anaerobes and *Proteus* for patients with chronic DFIs, which is easily ignored in clinical practice. In the present study, all samples were aerobically and anaerobically cultured. However, we did not use a special anaerobic container to transport the swab or tissue specimens in our hospital, which may be one potential explanation for the negative results of aerobic culture. On the other hand, the most representative bacterial taxa in intact skin included *Bacillales*, *Staphylococcaceae*, *Staphylococcus*, *Bacilli*, and *Actinobacteria*. Among them, previous research (Grice and Segre, 2011) has shown that *Staphylococcus epidermidis* (belonging to *Staphylococcus*, *Staphylococcaceae*, *Bacillales*, *Bacilli*, and *Firmicutes*) can bind keratinocyte receptors and inhibit the adherence of virulent *Staphylococcus aureus*. *Propionibacterium acnes* (belonging to *Propionibacterium*, *Propionibacteriaceae*, and *Actinobacteria*) can release fatty acids from lipid breakdown, acidifying the milieu, and inhibiting the growth of *Streptococcus pyogenes*. These symbiotic bacteria exist on the skin surface to maintain microecological balance, indicating that maintaining microecological balance may be an important way to prevent the occurrence of skin ulcers in diabetes. Moreover, there may be some “probiotics” that could promote wound healing at the skin surface. However, further study is required to prove this speculation.

In summary, PGM 16S rRNA sequencing technology is less time-consuming and a potential technique for the rapid microbiological diagnosis of DFIs. Ulcer swabbing which is relatively noninvasive and easy to perform, may be a suitable method for sampling bacterial pathogens in DFIs using the Levine technique, followed by 16S rRNA sequencing. Moreover, Gram-negative obligate anaerobes play a crucial role in DFIs, and opportunistic pathogens from the skin can lead to wound infections. The empirical use of broad-spectrum antibiotics covering Gram-negative obligate anaerobes should be considered for the treatment of moderate or severe DFIs.

## Limitations

The major limitations of this study include the small number of included patients and the fact that all DFUs were classified as PEDIS grade 3 or 4. Because of these limitations, our study did not analyze the bacterial communities in wounds at varying depths. In addition, some defects in PGM 16S rRNA gene sequencing technology have been noted: (1) potential bias in estimates of bacterial diversity may exist because many copies of the 16S rRNA gene are present in some species; (2) the selection of the 16S rRNA gene amplification area may affect the identification of bacterial species; and (3) the specific information at the species level cannot be obtained accurately because of the limited information available from selective amplification of the target gene fragment, and bacteria were only able to be identified to the genus level in this study. Despite the shortcomings of 16S rRNA gene sequencing technology, this technology has great potential for clinical microbiological diagnosis and research on microbial diversity with the development of new molecular biological techniques.

## Data availability statement

Raw sequence reads for this project have been deposited in the NCBI Sequence Read Archive (SRA) under the BioProject accession number PRJNA862325.

## Ethics statement

The studies involving human participants were reviewed and approved by the Medical Ethics Committee of Nanfang Hospital (No. NFEC-2015-104). The patients/participants provided their written informed consent to participate in this study.

## Author contributions

YH, ZX, and YC contributed equally to this study. YH performed the laboratory experiments and was responsible for writing the original draft. YC and ZX analyzed the data and reviewed the initial draft. MZ and YX performed the literature search and designed the study. YJ and XL collected the specimens. FG and YF designed the study and provided a critical review of the manuscript. All authors contributed to the article and approved the submitted version.

## Funding

This work was partially supported by a grant from the Wuhan Health Research Fund (grant number: WX19Y06).

## Conflict of interest

The authors declare that the research was conducted in the absence of any commercial or financial relationships that could be construed as a potential conflict of interest.

## Publisher’s note

All claims expressed in this article are solely those of the authors and do not necessarily represent those of their affiliated organizations, or those of the publisher, the editors and the reviewers. Any product that may be evaluated in this article, or claim that may be made by its manufacturer, is not guaranteed or endorsed by the publisher.
